# Clinical and genetic study of 12 Chinese Han families with nonsyndromic deafness

**DOI:** 10.1002/mgg3.1177

**Published:** 2020-02-12

**Authors:** Di Wu, Weiyuan Huang, Zhenhang Xu, Shuo Li, Jie Zhang, Xiaohua Chen, Yan Tang, Jinhong Qiu, Zhixia Wang, Xuchu Duan, Luping Zhang

**Affiliations:** ^1^ Department of Otolaryngology Affiliated Hospital of Nantong University Nantong China; ^2^ Department of Radiology Affiliated Hospital of Nantong University Nantong China; ^3^ School of Life Science Nantong University Nantong China; ^4^ Department of Otolaryngology Xiangya Hospital Central South University Changsha China

**Keywords:** deafness, gene mutation, next‐generation sequencing (NGS), nonsyndromic, phenotype

## Abstract

**Background:**

Nonsyndromic hearing loss is clinically and genetically heterogeneous. In this study, we characterized the clinical features of 12 Chinese Han deaf families in which mutations in common deafness genes *GJB2*, *SLC26A4,* and *MT‐RNR1* were excluded.

**Methods:**

Targeted next‐generation sequencing of 147 known deafness genes was performed in probands of 10 families, while whole‐exome sequencing was applied in those of the rest two.

**Results:**

Pathogenic mutations in a total of 11 rare deafness genes, *OTOF*, *CDH23*, *PCDH15*, *PDZD7*, *ADGRV1*, *KARS*, *OTOG*, *GRXCR2*, *MYO6*, *GRHL2*, and *POU3F4*, were identified in all 12 probands, with 16 mutations being novel. Intrafamilial cosegregation of the mutations and the deafness phenotype were confirmed by Sanger sequencing.

**Conclusion:**

Our results expanded the mutation spectrum and genotype‒phenotype correlation of nonsyndromic hearing loss in Chinese Hans and also emphasized the importance of combining both next‐generation sequencing and detailed auditory evaluation to achieve a more accurate diagnosis for nonsyndromic hearing loss.

## Graphical abstract

The 12 probands in our study have different phenotypes of hearing loss. Pathogenic mutations in a total of 11 rare deafness genes, OTOF, CDH23, PCDH15, PDZD7, ADGRV1, KARS, OTOG, GRXCR2, MYO6, GRHL2, and POU3F4, were identified in all 12 probands, with 17 mutations being novel.

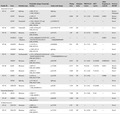

## 1 INTRODUCTION

Nonsyndromic hearing loss (NSHL) is one of the most common sensory defects in humans and is a remarkably complex and heterogeneous disease with variable phenotypes (Morton & Nance, 2006). Genetic components contribute significantly to the cause of hearing loss (http://hereditaryhearingloss.org), with mutations in a great variety of deafness genes being reported in the Chinese Han population (Hu et al., 2018; Sang et al., 2019; Yang, Wei, Chai, Li, & Wu, 2013; Zhang, Chai, Yang, & Wu, 2013; Zou et al., 2019). In recent years, next‐generation sequencing (NGS) technology including both targeted and whole‐exome sequencing has provided an easier and more cost‐effective approach for identifying causative mutations (Hu et al., 2018; Sang et al., 2019; Yang et al., 2013; Zhang et al., 2013; Zou et al., 2019). It provides crucial information for diagnosis, intervention, and treatment of hearing disorders (Zhang et al., 2013). In Chinese Hans, mutations in three genes, *GJB2* (121,011), *SLC26A4* (605,646), and *MT‐RNR1* (561,000), were commonly found in deaf patients, accounting for more than 30% of genetic causes of nonsyndromic deafness (Yang et al., 2013). In this light, we recruited a series of Chinese Han deaf families that were preexcluded from mutations in common deafness genes *GJB2,* *SLC26A4,* and *MT‐RNR1*. Targeted NGS for known deafness genes or whole‐exome sequencing (WES) were performed on the probands of each family to search for pathogenic mutations.

## 2 MATERIALS AND METHODS

### 2.1 Clinical evaluation

Patients from 12 deaf families were enrolled through the Department of Otolaryngology, Affiliated Hospital of Nantong University, Nantong, China. Comprehensive clinical evaluations, imaging examination results, audiograms, and other relevant clinical information were collected for the probands. All affected individuals were evaluated through detailed audiological evaluations as described previously (Hu et al., 2018; Zhang et al., 2013). The probands had no obvious syndromic symptoms other than the hearing loss. All subjects or their family members gave written, informed consent to participate in this study. This study was approved by the Ethics Committee of the Affiliated Hospital of Nantong University.

### 2.2 Genetic analysis

Genomic DNA from the family members were extracted from the blood samples using the Blood DNA kit (Tiangen Biotech). Prescreening of mutations in *GJB2,* *SLC26A4,* and *MT‐RNR1* was performed in all probands by Sanger sequencing. Among probands of the 12 deaf families, 10 were subjected to targeted NGS of 147 deafness‐related genes (File S1) and rest 2 to WES. Targeted gene capturing, data processing, bioinformatic analysis, and filtering against multiple databases for SNPs were performed as previously reported (Sang et al., 2019; Yang et al., 2013; Zou et al., 2019). For candidate pathogenic mutations, we filtered out: (a) all previously identified SNPs with allele frequencies of 0.005 or higher, (b) synonymous variants in the coding region, and (c) variants in the intronic or untranslated regions (with the exception of the splice site mutations or variants that may create an ectopic splice site). Intrafamilial cosegregate of the candidate variants and the deafness phenotype was confirmed by Sanger sequencing in all available family members (File S2).

## 3 RESULTS

### 3.1 Clinical manifestations

**Figure 1:**
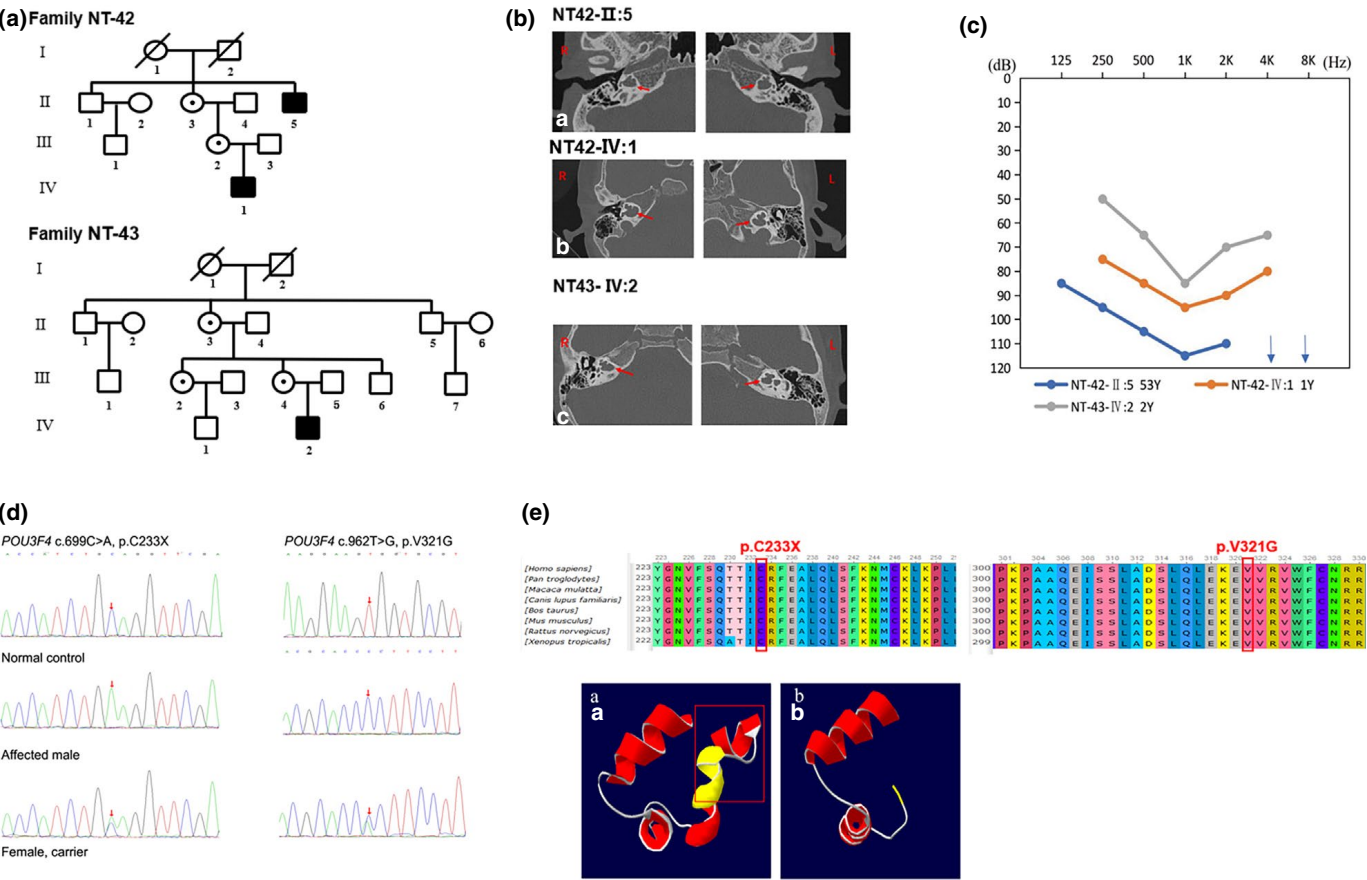
(a) Pedigree, (b) Temporal bone CT scan, (c) Audiograms, and (d, e) Mutation analysis in family NT42, NT‐43 [Correction added on 26 February 2020, after first online publication: In Figure 1 caption, the word ‘mutation’ in subfigure (d, e) has been capitalized so it reads ‘Mutation’.]

**Figure 2:**
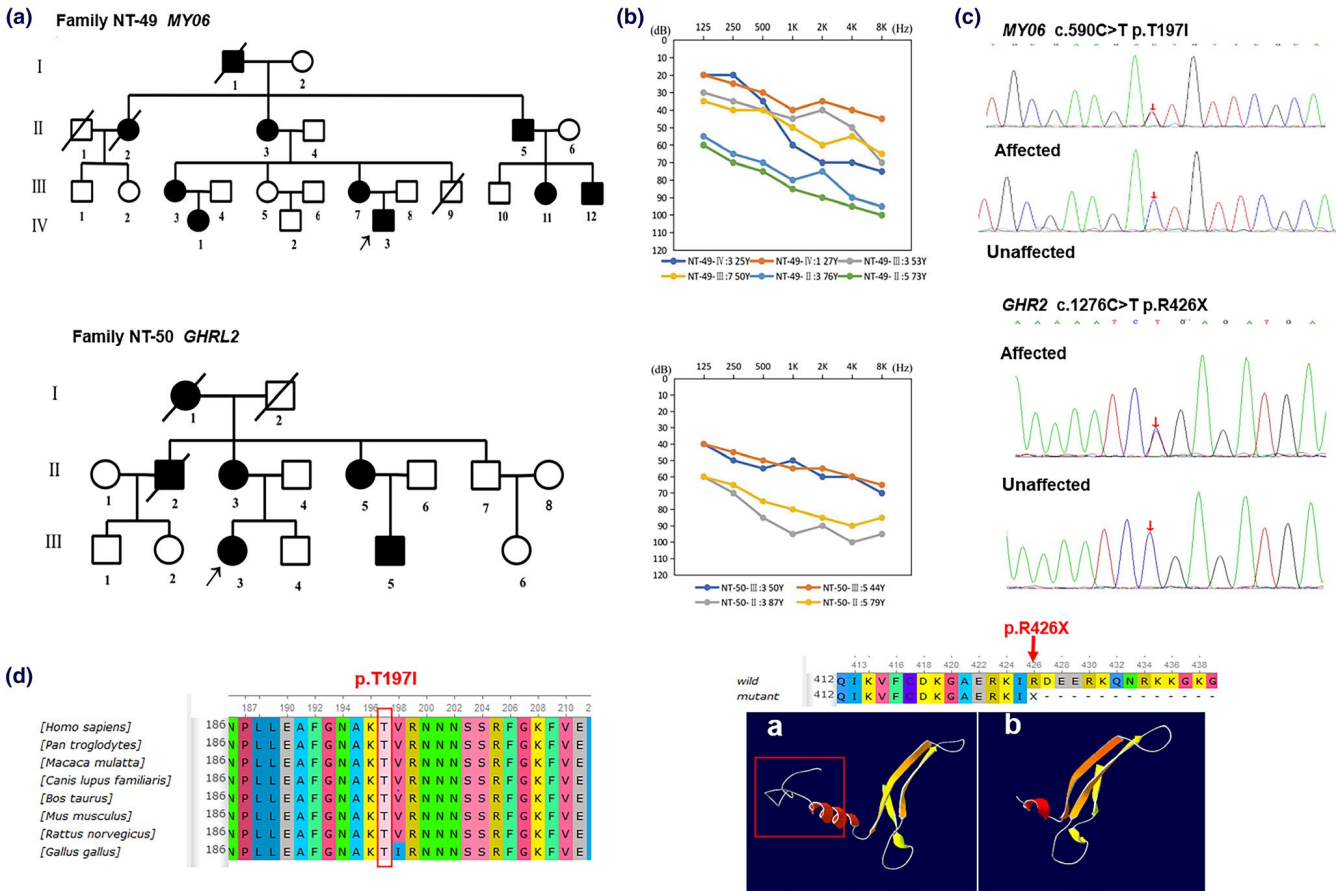
(a) Pedigree, (b) Audiograms, and (c, d) Mutation Analysis in family NT49, NT‐50 [Correction added on 26 February 2020, after first online publication: In Figure 2 caption, ‘Mutation Analysis’ has been inserted in subfigure (c, d).]

Patients in the 12 Chinese families, aged from 11 months to 87 years, exhibited bilateral, symmetrical, sensorineural hearing loss with variable developing course and degree of severity, ranging from stable to progressive and from mild to profound (Figures 1 and 2 and File S3). The age at onset of HL in these patients ranged from at birth to 44 years. A total of 12 families with NSHL were recruited in our study, including 6 simplex and 6 multiplex families. Through physical examination, no other abnormalities, such as retinal pigment degeneration or other optic defects, vestibular, neurologic, or systemic abnormalities, were detected in any of the patients, suggesting that the hearing loss is nonsyndromic. For family NT‐41, our audiological assessments revealed that proband NT‐41‐III:2 had congenital bilateral profound sensorineural hearing loss. Characteristic of auditory neuropathy spectrum disorder (ANSD), this patient lacked auditory brainstem response (ABR) in both ears while the distortion product otoacoustic emission (DPOAE) was present. At age 1‐year‐2‐month, the affected proband underwent left‐side cochlear implantation (CI). Three years after CI, the ANSD subject is enrolled in regular school with good post‐CI outcome, similar to our previous report (Zhang et al., 2013). Patients with X‐linked deafness in Families NT‐42 and NT‐43, carrying mutations in *POU3F4* (300,039) as subsequently revealed, showed characteristic inner ear radiological features (Figure 1) compatible with incomplete partition type3 (IP3), including absent modiolus and lamina spiralis but preserved interscalar septum in a normal‐sized cochlea and abnormal dilatation of the lateral end of the internal auditory canal (IAC). In family NT‐42, two male patients NT42‐IV:1 and NT42‐II:5 had congenital severe‐to‐profound sensorineural hearing loss. Similarly, proband NT‐43‐III:2 also exhibited severe sensorineural deafness. Female mutation carriers in the two families had completely normal hearing. Temporal bone CT images of the three patients revealed characteristic anomalies for IP3 with an increased risk of gusher during CI surgery. Patient NT‐42‐IV:1 received right‐side (CI) at the age of 1 year and 2 months. As expected, CSF gusher was seen while no complications related to surgery were observed. Three years after CI, the patient was enrolled in regular school with good CI outcome. Patient NT‐43‐III:2 showed slight progression in hearing loss after 3 years of follow‐up. The patient used a hearing aid with satisfactory effect.

### 3.2 Genetic findings

The 12 Chinese probands have been previously excluded for mutations in common deafness genes *GJB2,* *SLC26A4,* and *MT‐RNR1* by Sanger sequencing. To detect possible causative mutations by targeted NGS or WES, nonsynonymous variants with minor allele frequencies lower than 0.005 were filtered through as previously described (Hu et al., 2018; Sang et al., 2019; Yang et al., 2013; Zou et al., 2019). Candidate causative variants were summarized in Table S2. In eight recessive probands, bi‐allelic mutations, confirmed by parental genotyping, were identified in known deafness genes *OTOF* (603,681)*,* *CDH23* (601,067)*,* *PCDH15* (605,514)*,* *ADGRV1* (602,851)*,* *PDZD7* (612,971)*,* *KARS* (601,421)*,* *OTOG* (604,487), *and* *GRXCR2* (615,762) (*n* = 1 each, Table 1). In two dominant families, we identified two heterozygous variants in genes associated with dominant deafness, p.T197I in *MYO6* (600,970) and p.R426X in *GRHL2* (608,576), cosegregating with the hearing impairment (Figure 2). Consistent with X‐linked recessive inheritance pattern, in Families NT‐42 and NT‐43, we identified two hemizygous candidate mutations p.C233X and p.V321G in *POU3F4*, respectively (Table 1). Sanger sequencing in extended family members confirmed the cosegregation of the reported mutations with the hearing phenotype (Figures 1 and 2 and Files S3 and S4). Among the 19 mutations identified in this study, 16 mutations have not been associated with deafness in previous reports (Table 1) (Lee et al., 2014; Sloan‐Heggen et al., 2016; Yang et al., 2013). Two intronic variants, c.13893+8T>G in *ADGRV1* and c.2117‐6C>T in *OTOG* are further away from the splice sites. Although extremely low in MAF in the control population, the pathogenicity of these two variants is undetermined in this study.

**Table 1:** Mutations detected in 12 Chinese Han families

Family ID	Gene	Mutation type	Nucleotide change (Transcript version)	Amino acid change	Phylop score	Mutation taster	PROVEAN (score)	SIFT (score)	Allele frequency in controls	Novel or HGMD
Autosomal recessive										
NT‐41	*OTOF*	Splicing	c.4961‐3C>G （NM_194248）	Splicing	—	—	—	—	—	Known Pathogenic
	
	*OTOF*	Missense	c.145C>T (NM_194248)	p.R49W	8.066	DC	D (−3.38)	D (0.002)	0.0095	Known Benign
	
	*OTOF*	Frameshift	c.1364_1365AC>TT and c.1366_1367insC (NM_194248)	p.Y455Ffs*21	—	—	—	—	—	Novel
	
NT‐44	*CDH23*	Frameshift	c.9469_9470insGT (NM_022124)	p.E3158Vfs*58	4.707	DC	—	—	—	Novel
	
NT‐45	*PCDH15*	Missense	c.4310C>T (NM_033056)	p.P1437L	1.89	DC	N (−0.75)	T (0.081)	—	Novel
	
	*PCDH15*	Codon Mutation	c.5254_5280delCCTATTTCTCCTCCTTCTCCTCCTCCT (NM_033056)	p.1752_1760delPISPPSPPP	—	—	—	—	0.0001	Novel
	
NT‐46	*ADGRV1*	Missense	c.11411G>A (NM_032119)	p.R3804Q	8.61	DC	D (−3.21)	D (0)	—	Novel
	
	*ADGRV1*	Splicing	c.13893+8T>G (NM_032119)	splicing	—	—	—	—	—	Novel
	
NT‐47	*PDZD7*	Nonframeshift	c.1574_1597delACCAGGAGAGGGGCCGGGCCCTGC (NM_001195263）	p.525_533delDQERGRALLinsV	—	—	—	—	—	Novel
	
	*PDZD7*	Missense	c.490C>T (NM_001195263)	p.R164W	0.653	DC	D (−6.05)	D (0.008)	0.00005283	Novel
	
NT‐48	*KARS*	Missense	c.685T>C (NM_001130089)	p.Y229H	0.277	PO	N (0.36)	T (0.593)	0.0011	Known Benign
	
	*KARS*	Missense	c.403G>A (NM_001130089)	p.D135N	3.049	DC	D (−2.26)	T (0.241)	—	Novel
	
NT‐51	*OTOG*	Missense	c.433G>A (NM_001277269)	p.G145S	9.516	DC	D (−4.95)	—	0.0006	Novel
	
	*OTOG*	Splicing	c.2117‐6C>T (NM_001277269)	Splicing	—	—	—	—	—	Novel
	
NT‐52	*GRXCR2*	Missense	c.65A>G （NM_001080516）	p.K22R	3.254	DC	D (−2.61)	D (0.006)	—	Novel
	
Autosomal dominant										
NT‐49	*MYO6*	Missense	c.590C>T （NM_004999）	p.T197I	7.568	DC	D (−5.84)	D (0)	—	Novel
	
NT‐50	*GRHL2*	Nonsense	c.1276C>T (NM_024915)	p.R426X	1.858	DC	—	—	—	Novel
X‐linked recessive										
NT‐42	*POU3F4*	Nonsense	c.699C>A (NM_000307)	p.C233X	3.78	DC	—	—	—	Novel
	
NT‐43	*POU3F4*	Missense	c.962T>G (NM_000307)	p.V321G	6.105	DC	D (−6.95)	D (0)	—	Novel
	

Abbreviations: D, deleterious; N, neutral; T, tolerated; DC, Disease causing; PO, Polymorphism.

## 4 DISCUSSION

In this study, we performed a detailed clinical and genetic characterization of 12 Chinese Han families affected by autosomal recessive, autosomal dominant, and X‐linked NSHL. In family NT‐41, bi‐allelic candidate variants in *OTOF*, a gene associated with ANSD, were identified by targeted NGS. Interestingly, three different variants in *OTOF* were identified in this patient. Among them, c.4961‐3C>G and p.R49W have been previously reported to be associated with nonsyndromic deafness (Sloan‐Heggen et al., 2016; Yang et al., 2013) while p.R49W was already reported as Benign (ClinVar, Deafness Variation Database), however, were inherited from the same maternal allele, suggesting that c.4961‐3C>G should be pathogenic. Similarly, the continuous variant, c.1364_1365AC>TT and c.1366_1367insC were inherited from the same paternal allele. Considering it introduces a frameshifting variant p.Y455Ffs*21 in *OTOF*, it is probably the true pathogenic mutation. In accordance with the report by He et al. (2018), our data suggested that targeted NGS accompanied by parental genotyping provides a simple but effective step toward minimizing the false‐positive results.

Mutations in *CDH23* and *PCDH15* may lead to both NSHL (DFNB12 and DFNB23, respectively) and Usher syndrome type 1 (USH1D and USH1F, respectively) characterized by both congenital hearing loss and childhood retinitis pigmentosa (Astuto et al., 2002; Hu et al., 2018; Zhan, Liu, & Chen, 2015). Proband NT‐44‐II:1 carried a homozygous c.9469_9470insGT mutation in *CDH23*. The parents of this patient, although not consanguineously married, each carried a heterozygous mutation and are likely distally related. Proband NT‐44‐II:1 was 11 months old at the time of test when congenital profound sensorineural HL was diagnosed. At the age of 13 months, the patient underwent right‐side CI. Followed up until 4 years old, the patient showed good speech and language recognition. Although no ophthalmologic abnormalities were observed, we cannot definitely rule out the possibility that this young patient may develop retinopathy later in the life. In combination with our previous study (Hu et al., 2018), we identified a relatively high prevalence (4/22) of *CDH23* mutations in Chinese Han deaf patients, and our reports of these novel mutations expanded the *CDH23* mutation spectrum. For family NT‐45, the two affected siblings NT45‐II:1 and NT45‐II:2 carried compound heterozygous mutations p.P1437L/p.1752_1760del in *PCDH15.* The two patients, aged 39 and 49, respectively, at the time of test, showed slowly progressive and moderate hearing loss with onset between 25 and 30 years of age. Troublesome tinnitus was also reported for both, while no ophthalmologic abnormalities were observed, supporting that mutations in *PCDH15* cause not only USH1F but also DFNB23 (Astuto et al., 2002; Zhan et al., 2015).

In family NT‐46, we detected compound heterozygous mutations p.R3804Q/c.13893+8T>G in *ADGRV1*, which segregated with hearing loss in this family. Both mutations are novel. The age of two affected siblings NT‐46‐II:3 and NT‐46‐II:6 were 67 and 61, respectively. Both patients experienced moderate and slowly progressive hearing loss with onset between 30 and 35 years and suffered from tinnitus. No signs of visual or vestibular disorder were observed. Mutations in *ADGRV1* may result in Usher syndrome 2C, which is characterized by congenital moderate‐to‐severe hearing loss, retinal degeneration in the second decade of life or later, and normal vestibular function (Zhang, Wang, Liu, Liu, & Jiang, 2018). To date, only two previous reports have associated this gene for NSHL (Sang et al., 2019; Yang et al., 2013), but the affected patients in those reports may be too young to present signs of retinal degeneration and vestibular dysfunction. In contrast, our cases had milder and progressive HL, yet at the age of over 60 did not have any vision problems, which may further validate *ADGRV1* in association with NSHL. For Family NT‐47, we identified novel compound heterozygous mutations p.525_533delDQERGRALLinsV/p.R164W in *PDZD7*. Previous report has associated *PDZD7* mutations with digenic Usher syndrome (Ebermann et al., 2010) and DFNB57 (Guan et al., 2018; Luo et al., 2019). To our knowledge, our study is the third report to identify *PDZD7* as a causative gene for autosomal recessive nonsyndromic hearing loss (ARNSHL) in the Chinese population (Guan et al., 2018; Luo et al., 2019). Patient NT‐47‐II:1 failed the newborn hearing screening by automated auditory brainstem response (AABR) and had congenital moderate sensorineural hearing loss confirmed by ABR and audio steady‐state response (ASSR) at 2 years of age. After a 3‐year follow‐up, patient NT46‐II:2 had no signs of visual or vestibular disorder and no obvious progression in hearing loss at age of 5. The patient was enrolled in a regular school with hearing aids. For Family NT‐48, one novel variant p.D135N and one known variant p.Y229H were identified in *KARS*, which encodes lysyl‐tRNA synthetase (LysRS), as the only candidate causative variants (Santos‐Cortez et al., 2013; Lee et al., 2014). The affected Individual NT‐48‐II:5 was a 47‐year‐old female with severe hearing impairment affecting primarily higher frequencies. She experienced progressive hearing loss with onset between 10 and 15 years of age. No other systemic abnormalities were detected.

We also ascertained two Chinese families with an autosomal dominant form of progressive NSHL. Mutations in *MYO6* have been associated with dominant and recessive nonsyndromic hearing loss DFNA22 and DFNB37 (Ahmed et al., 2003; Kwon et al., 2014). The hearing loss in affected members of Family NT‐49 was progressive, midlife onset, and mild to severe, affecting high frequencies to the greatest degree. The hearing impairment gradually progressed to all frequencies later and eventually became severe in the seventh decade. To date, only two mutations in *GRHL2* have been described (Peters et al., 2002; Vona, Nanda, Neuner, Müller, & Haaf, 2013). Phenotypic characterization of Family NT‐50 shows that the p.R426 X mutation in *GRHL2* resulted in progressive, bilateral hearing loss with a typical onset in middle adulthood, which was consistent with the phenotype reported for the other two DFNA28 families (Peters et al., 2002; Vona et al., 2013). Our clinical data supported the emerging genotype‒phenotype correlation for DFNA22 and DFNA28.

In this study, our targeted NGS analysis identified mutations in nine rare deafness genes in aforementioned 10 families. In recent years, whole‐exome sequencing (WES) has become a powerful tool for both new gene discovery and molecular diagnosis in hereditary hearing loss (Sang et al., 2019; Zou et al., 2019). Here we used proband‐WES approach to successfully identify novel compound heterozygous mutations p.G145S/c.2117‐6C>T in *OTOG* and a homozygous p.K22R mutation in *GRXCR2* in Family NT‐51 and NT‐52, respectively. To our knowledge, this is the first reported *OTOG* mutation associated with hearing loss in China (Danial‐Farran et al., 2018; Yu et al., 2019). In Family NT‐51, the patient had experienced progressive and steeply sloping high‐frequency hearing loss without any vestibular impairment. She also reported troublesome tinnitus. *GRXCR2* mutations are rare causes of recessive deafness as there is only one report worldwide (Imtiaz, Kohrman, & Naz, 2014). The 72‐year‐old proband in NT‐52 had a moderate sensorineural hearing loss affecting primarily high frequencies, resulting in a downsloping audiometric configuration. Her hearing loss started during her mid‐40s and followed by steady and gradual progression. The proband had a less severe hearing loss as compared to previous study (Imtiaz et al., 2014), suggesting a variable genotype‒phenotype correlation.

## 5 CONCLUSION

In this report, we performed a comprehensive mutation screening by targeted NGS or WES in 12 Chinese families with NSHL. Our results revealed a number of novel or recurrent mutations in rare deafness genes and supported the heterogeneity of the genetic and phenotypic spectrum of NSHL in Chinese Hans. Our study also showed that combining NGS‐based molecular diagnosis and detailed clinical evaluation can achieve a more accurate diagnosis for NSHL patients.

## CONFLICT OF INTEREST

The authors declare no conflict of interest.

## Data Availability

The data relating to the findings of this study are available from the corresponding author.
