# Supplementary material for: Clinical and genetic study of 12 Chinese Han families with nonsyndromic deafness
Source: Mol Genet Genomic Med. 2020 Feb 12;8(4):e1177. doi: 10.1002/mgg3.1177 (PMC7196461; doi:10.1002/mgg3.1177)
Supplement: Supplementary file 1 [file MGG3-8-e1177-s001.pdf]

Supplementary material file 1: Table S1 147 genes targeted for the next-generation sequencing

ACTG1[N]	COL9A1[S]	FGF8[S]	ILDR1[N]	MYH9[N/S]	PROK2[S]	SOX10[S]
ALX3[S]	COL9A2[S]	FGFR1[S]	KARS[N/S]	MYO15A[N]	PROKR2[S]	STRC[N]
ADGRV1[N/S]	COMT2[S]	FGFR3[S]	KCNE1[S]	MYO1A[N]	PRPS1[S]	TBC1D24[N/S]
BSND[S]	CRYM[N]	FLNA[S]	KCNJ10[N/S]	MYO3A[N]	PTPRQ[N]	TCOF1[S]
CABP2[N]	DFNA5[N]	FOXI1[S]	KCNQ1[S]	MYO6[N]	RDX[N]	TECTA[N]
CCDC50[N]	DFNB31[N/S]	FREM1[S]	KCNQ4[S]	MYO7A[N/S]	RPGR[S]	THOC1[N]
CDH23[N/S]	DFNB59[N]	GATA3[S]	KITLG[S]	NDP[S]	SALL1[S]	TIMM8A[S]
CEACAM16[N]	DIABLO[N]	GIPC3[N]	KRT9[S]	NF2[N]	SALL4[S]	TJP2[N]
CHD7[S]	DIAPH1[N]	GJB2[N/S]	LAMA3[S]	OTOA[N]	SANS[S]	TMC1[N]
CIB2[N/S]	DIAPH3[N]	GJB3[N/S]	LARS2[S]	OTOF[N]	SEC23A[S]	TMIE[N]
CLDN14[N]	DNAJC17 [N]	GJB6[N]	LHFPL5[N]	OTOG[N]	SEMA3E[S]	TMPRSS3[N]
CLPP[S]	DSPP[S]	GPSM2[S]	LOXHD1[N]	P2RX2[N]	SERPINB6[N]	TNC[N]
CLRN1[S]	ECM1[U]	GRHL2[N]	LRTOMT[N]	PABPN1[S]	SIX1[N/S]	TPRN[N]
COCH[N/S]	EDN3[S]	GRXCR1[N]	GRXCR2[N]	PAX3[S]	SIX5[S]	TRIOBP[N]
COL11A1[N/S]	EDNRB[S]	HARS[S]	MARVELD2[N]	PCDH15[N/S]	SLC17A8[N]	TRMU[S]
COL11A2[N/S]	ELMOD3[N]	HARS2[S]	miR-96[N]	PDZD7[S]	SLC19A2[N]	TSPEAR[N]
COL2A1[S]	ESPN[N]	HGF[N]	miR-182[N]	PNPT1[N]	SLC26A4[N/S]	USH1C[S]
COL4A3[S]	ESRRB[N]	HMX1[S]	miR-183[S]	POLR1C[S]	SLC26A5[N]	USH1G[S]
COL4A4[S]	EYA1[S]	HOXA2[S]	MITF[S]	POLR1D[S]	SMAD4[S]	USH2A[S]
COL4A5[S]	EYA4[N/S]	HSD17B4[S]	MSRB3[N]	POU3F4[N]	SMPX[N/S]	WFS1[N/S]
COL4A6[N/S]	FGF3[S]	IL13[S]	MYH14[N/S]	POU4F3[N]	SNAI2[S]	WHRN[N/S]

[N] Genes for non-syndromic hearing loss; [S] Genes for syndromic hearing loss
