# Supplementary material for: Clinical and genetic study of 12 Chinese Han families with nonsyndromic deafness
Source: Mol Genet Genomic Med. 2020 Feb 12;8(4):e1177. doi: 10.1002/mgg3.1177 (PMC7196461; doi:10.1002/mgg3.1177)
Supplement: Supplementary file 2 [file MGG3-8-e1177-s002.pdf]

Supplementary material file 2: Table S2 All variants identified by targeted NGS or proband whole-exome sequencing

**Proband**	**Gene**	**Ref_number**	**Nucletide change**	**Amino acid change**	**Genotype**	**Allele frequency in controls**	**Cosegragation with the deafness**
**NT-41**	*OTOF*	NM_194248	c.4961-3C>G	Splicing	Heterozygous	0/600	Yes
			c.1364_1365AC>TT and c.1366_1367insC	p.V455Ffs*21	Heterozygous	0/600	Yes
			c.145C>T	p.R49W	Heterozygous	0/600	Yes
	GJB3	NM_024009	c.580G>A	p.A194T	Heterozygous	0/600	No
	USH2A	NM_206933	c.10855G>A	p.V3619I	Heterozygous	0/600	No
**NT-42**	*POU3F4*	NM_000307	c.699C>A	p.C233X	Hemizygous	0/600	Yes
	EYA1	NM_000503	c.452G>C	p.G151A	Heterozygous	0/600	No
	MYO15A	NM_016239	c.9478C>T	p.L3160F	Heterozygous	1/600	No
**NT-43**	*POU3F4*	NM_000307	c.962T>G	p.V321G	Hemizygous	0/600	Yes
	MYO7A	NM_000260	c.2485G>C	p.V829L	Heterozygous	0/600	No

	KCNQ4	NM_004700	c.546C>G	p.F182L	Heterozygous	1/600	No
	CDH23	NM_022124	c.7466G>A	p.R2489H	Heterozygous	0/600	No
	LOXHD1	NM_144612	c.4147A>G	p.T1383A	Heterozygous	0/600	No
**NT-44**	*CDH23*	NM_022124	c.9469_9470 insGT	p.E3158Vfs*58	Homozygous	0/600	Yes
	KARS	NM_001130089	c.49A>T	p.T17S	Heterozygous	0/600	No
	TRIOBP	NM_001039141	c.536G>T	p.R179L	Heterozygous	0/600	No
**NT-45**	*PCDH15*	NM_033056	c.4310C>T	p. P1437L	Heterozygous	0/600	Yes
			c.5254_5280 delCCTATTT CTCCTCCT TCTCCTCC TCCT	p.1752_1760 delPISPPSP PP	Heterozygous	1/600	Yes
	MYO1A	NM_005379	c.2972C>T	p.T991M	Heterozygous	0/600	No
	USH2A	NM_206933	c.5554C>A	p.H1852N	Heterozygous	0/600	No
	TNC	NM_002160	c.3124G>T	p.G1042C	Heterozygous	0/600	No

**NT-46**	*ADGRV1*	NM_032119	c.11411G>A	p.R3804Q	Heterozygous	0/600	Yes
			c.13893+8T >G	splicing	Heterozygous	0/600	Yes
	LRTOMT	NM_001145308	c.593C>T	p.P198L	Heterozygous	0/600	No
	TRIOBP	NM_001039141	c.1195C>G	p.R399G	Heterozygous	1/600	No
	USH2A	NM_206933	c.10388A>G	p.D3463G	Heterozygous	0/600	No
**NT-47**	*PDZD7*	NM_001195263	c1574_1597 delACCAGG AGAGGGGC CGGGCCCT GC	p.525_533 delDQERGR ALLinsV	Heterozygous	0/600	Yes
			c.490C>T	p.R164W	Heterozygous	0/600	Yes
	SERPINB6	NM_004568	c.859C>T	p.R287C	Heterozygous	0/600	No
**NT-48**	*KARS*	NM_001130089	c.685T>C	p.Y229H	Heterozygous	1/600	Yes
			c.403G>A	p.D135N	Heterozygous	0/600	Yes
	CDH23	NM_022124	c.7384T>C	p.S2462P	Heterozygous	0/600	No

	COL4A4	NM_000092	c.1379G>A	p.C460Y	Heterozygous	0/600	No
	ESPN	NM_031475	c.315_316 delCA	p.T106Sfs*57	Heterozygous	0/600	No
	LOXHD1	NM_144612	c.4486C>T	p.L1496F	Heterozygous	0/600	No
**NT-49**	*MYO6*	NM_004999	c.590C>T	p.T197I	Heterozygous	0/600	Yes
	PCDH15	NM_033056	c.5156T>A	p.I1719N	Heterozygous	0/600	No
	ADGRV1	NM_032119	c.7071G>A	p.M2357I	Heterozygous	0/600	No
	BSND	NM_057176	c.893G>A	p.G298E	Heterozygous	0/600	No
	SLC19A2	NM_006996	c.1213A>G	p.T405A	Heterozygous	0/600	No
**NT-50**	*GRHL2*	NM_024915	c.1276C>T	p.R426X	Heterozygous	0/600	Yes
	USH2A	NM_206933	c.5608C>T	p.R1870W	Heterozygous	0/600	No
**NT-51**	*OTOG*	NM_001277269	c.433G>A	p.G145S	Heterozygous	0/600	Yes
			c.2117-6C>T	splicing	Heterozygous	0/600	Yes
	KCNQ4	NM_004700	c.1421C>T	p.T474I	Heterozygous	0/600	No

	PIEZO1	NM_001142864	c.5743C>T	p.R1915C	Heterozygous	1/600	No
		NM_001142864	c.5482G>C	p.E1828Q	Heterozygous	0/600	No
**NT-52**	*GRXCR2*	NM_001080516	c.65A>G	p.K22R	Homozygous	0/600	Yes
	CUBN	NM_001081	c.8968G>A	p.V2990I	Heterozygous	0/600	No
	HYDIN	NM_001270974	c.11712delT	p.Q3905Rfs*4	Heterozygous	0/600	No
	MEFV	NM_000243	c.1105C>T	p.P369S	Heterozygous	0/600	No
