# Supplementary material for: Clinical and genetic study of 12 Chinese Han families with nonsyndromic deafness
Source: Mol Genet Genomic Med. 2020 Feb 12;8(4):e1177. doi: 10.1002/mgg3.1177 (PMC7196461; doi:10.1002/mgg3.1177)

Supplementary material file 3: Pedigrees and Audiograms of **NT-41～48,51-52**.

Autosomal Recessive（8/12）

Family NT-41 *OTOF*

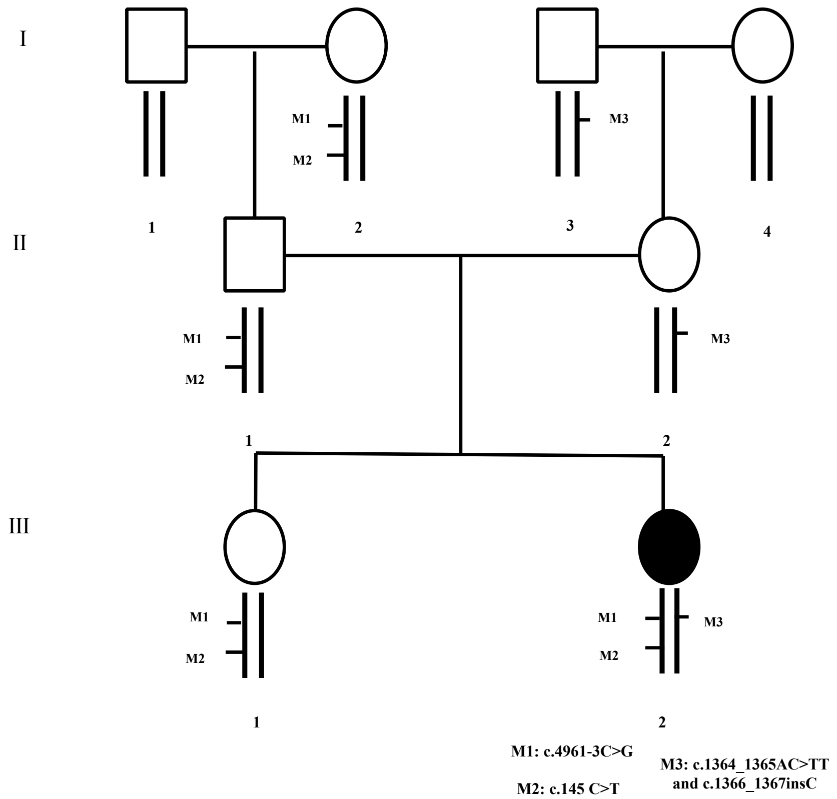

Family NT-44 *CDH23*

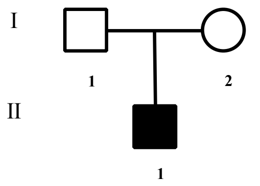

Family NT-45 *PCDH15*

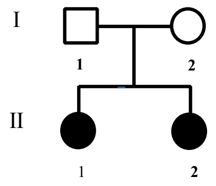

Family NT-46 *ADGRV1*

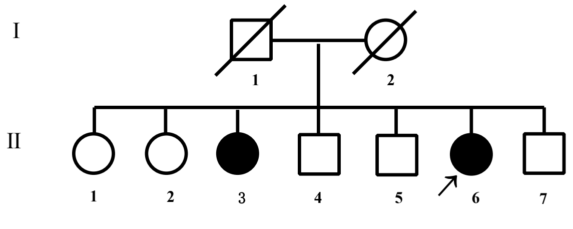

Autosomal Recessive（8/12）

Family NT-47 ***PDZD7***

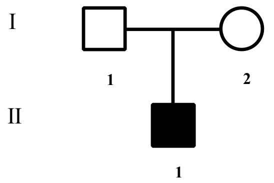

Family NT-48 ***KARS***

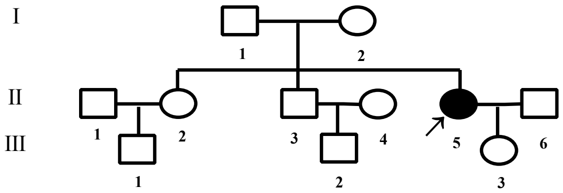

Family NT-51 ***OTOG***

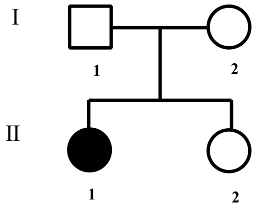

Family NT-52 ***GRXCR2***

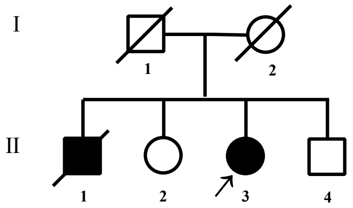

Audiograms(ASSR) for NT-41～44,47(A)

Audiograms for NT-45,46,48,51,52(B)

A

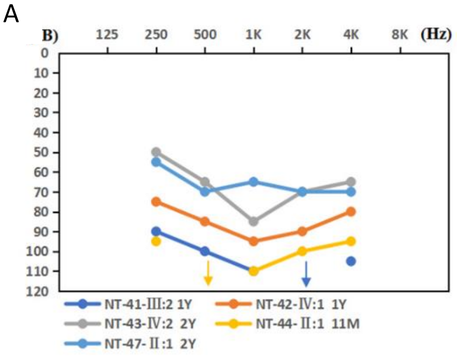

B

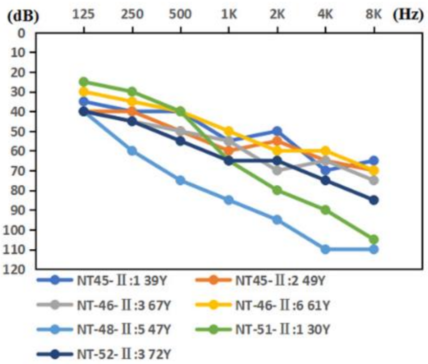

Supplement: Supplementary file 3 [file MGG3-8-e1177-s003.pdf]
