# Supplementary material for: Clinical and genetic study of 12 Chinese Han families with nonsyndromic deafness
Source: Mol Genet Genomic Med. 2020 Feb 12;8(4):e1177. doi: 10.1002/mgg3.1177 (PMC7196461; doi:10.1002/mgg3.1177)
Supplement: Supplementary file 4 [file MGG3-8-e1177-s004.pdf]

Supplementary material file 4: Validation of candidate mutations by PCR-Sanger sequencing and in silico function analysis on candidate mutations. Evolutionary conservation and structure of wild-type and mutant-type created by SWISS-MODEL.

Autosomal Recessive（8/12）

NT41-III:2（F/1Y）

OTOF c.145C>T, p.R49W

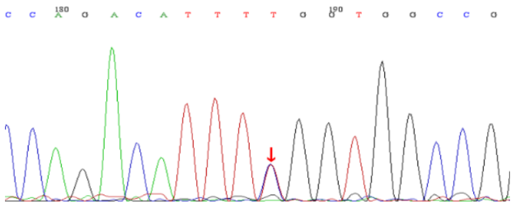

OTOF c.4961-3C>G

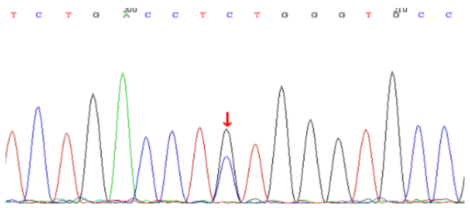

OTOF c.1364_1365AC>TT and c.1366_1367insC, p.Y455Ffs*21

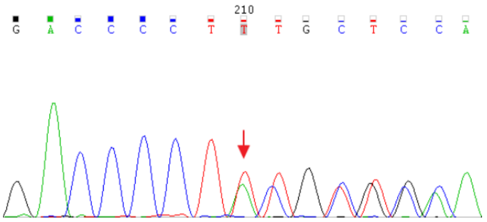

（The 5’ to 3’ strand sequence）

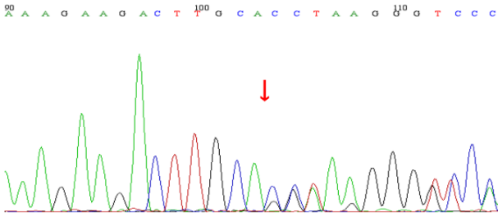

（The reverse sequence）

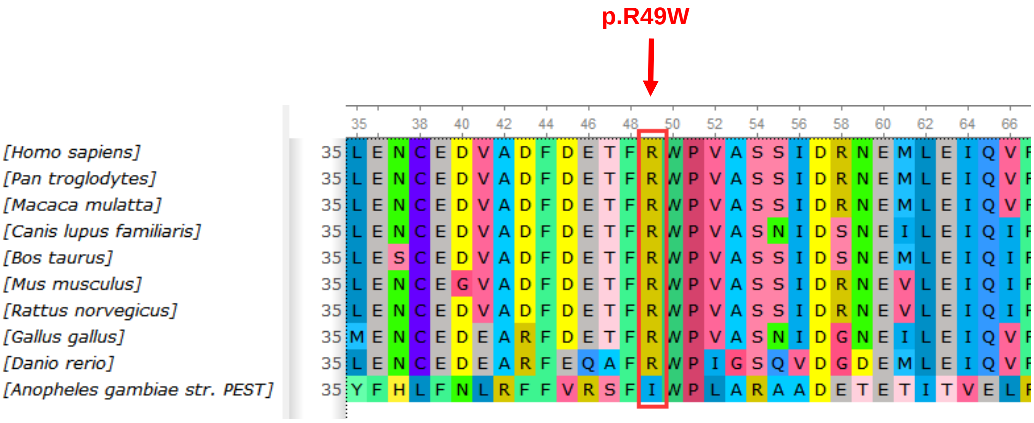

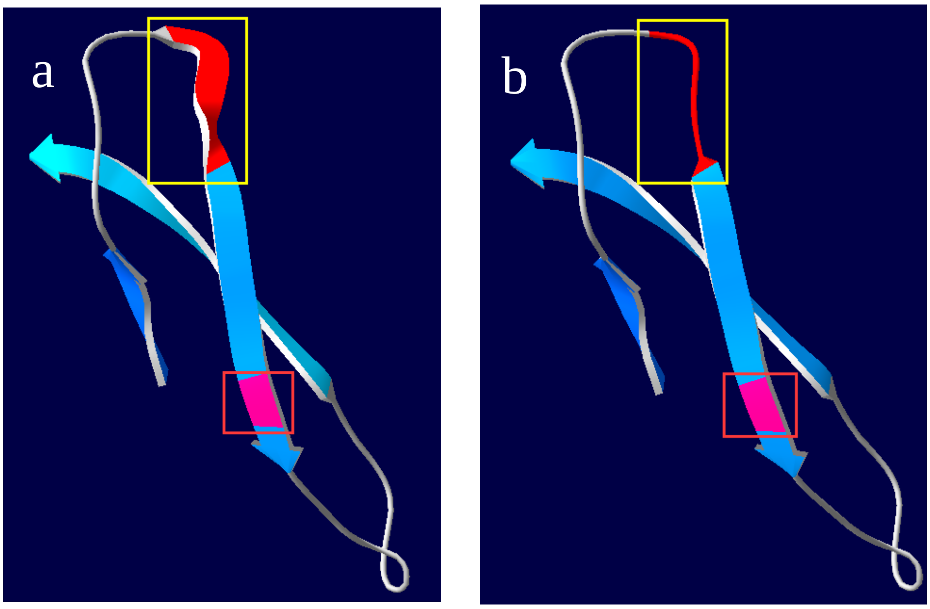

Wild（a），Mutation p.R49W（b）: A hydrogen bond between 34 and 36 was broken and two hydrogen bonds were formed among 41 and 43, 43 and 44 after the 49 Trp mutation.

OTOF p.Y455Ffs*21

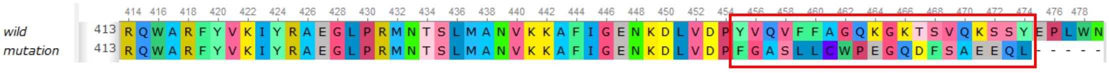

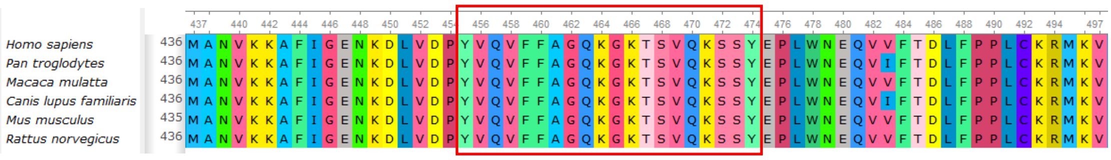

There is no protein structural homology-model for p.Y455Ffs*21.

NT44-II:1（M/11Months）

CDH23 c.9469_9470insGT, p.E3158Vfs*58

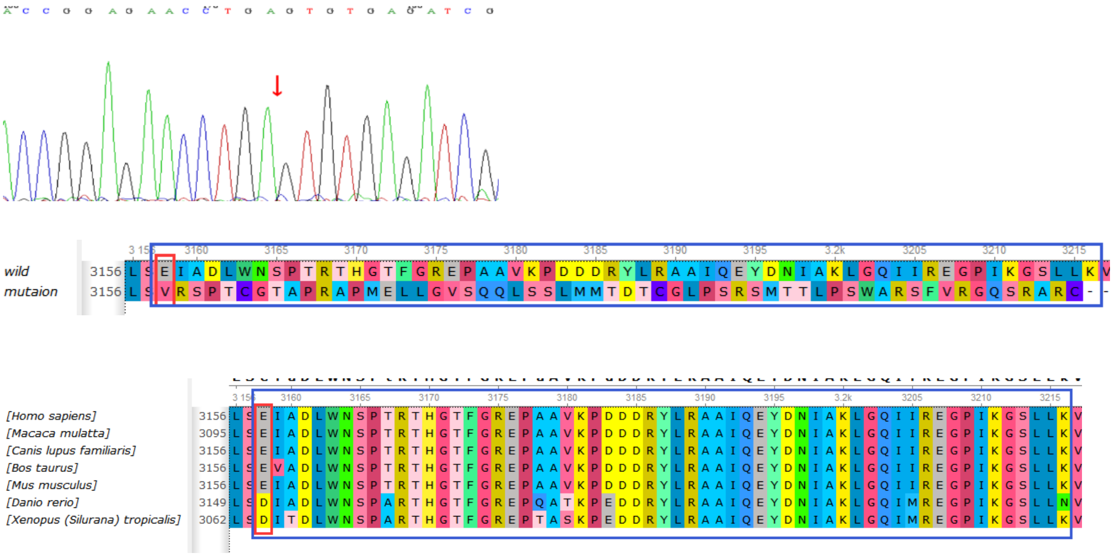

There is no protein structural homology-model for p.E3158Vfs*58.

NT45-II:1（F/39Y）

PCDH15 c.4310C>T, p.P1437L *PCDH15* c.5254_5280del, p.1752_1760del

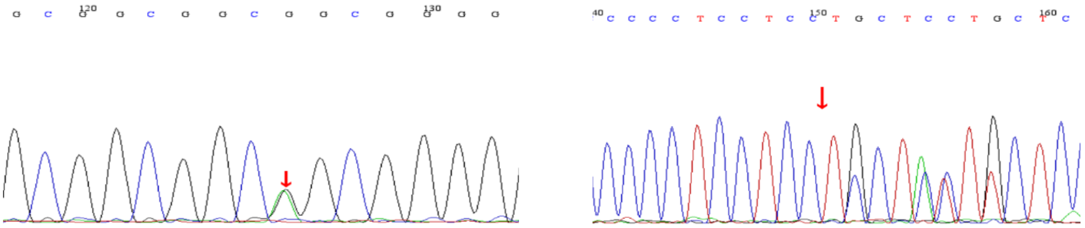

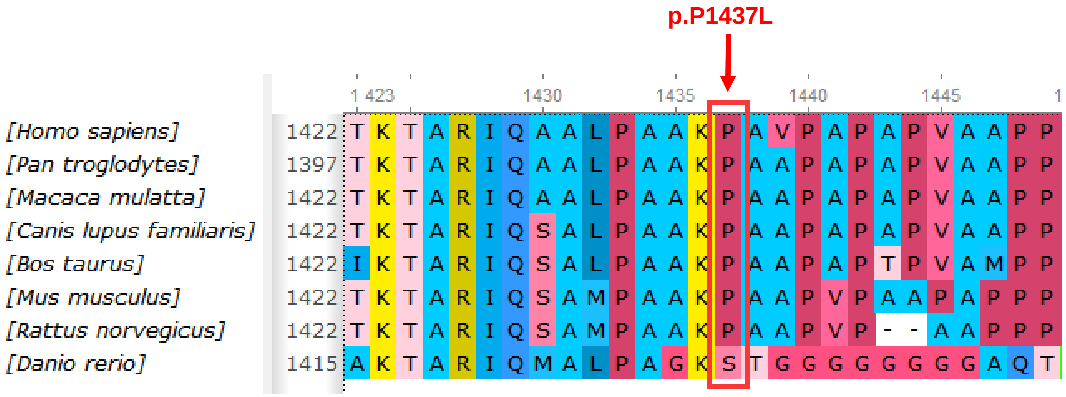

There is no protein structural homology-model for p.P1437L.

Mutation (p.1752_1760delPISPPSPPP)

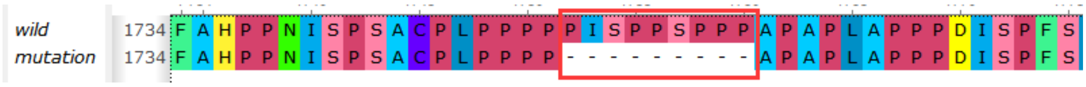

There is no protein structural homology-model for p.1752_1760delPISPPSPPP.

NT46-II:6（F/61Y）

ADGRV1 c.11411G>A, p.R3804Q *ADGRV1* c.13893+8T>G

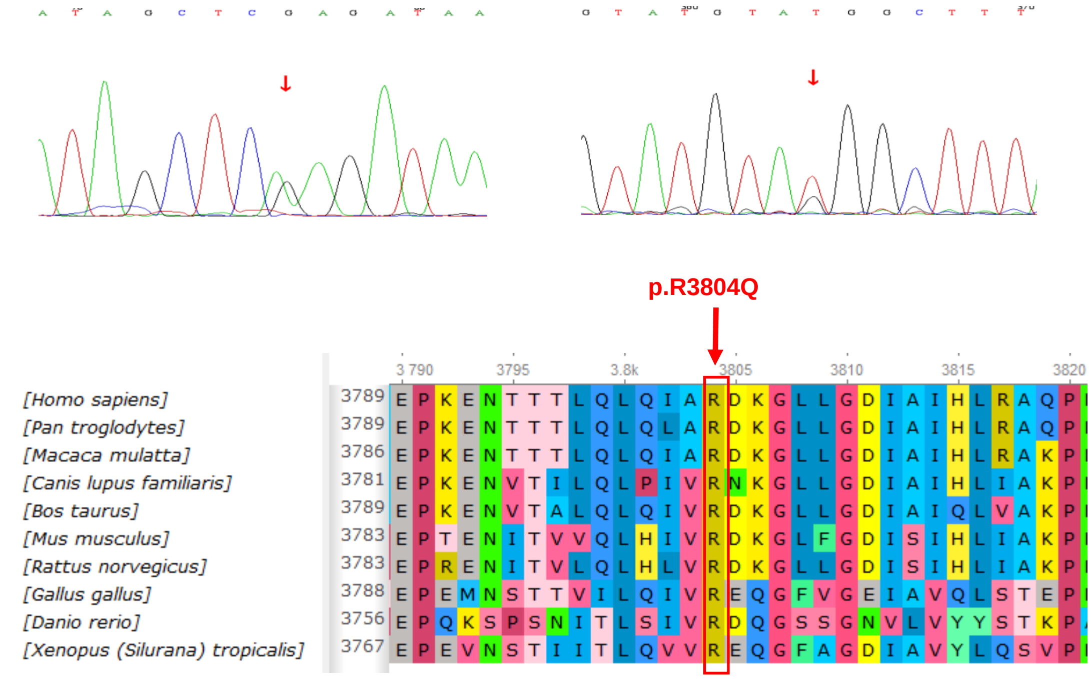

There is no protein structural homology-model for p.R3804Q.

NT-47-II:1（M/2Y）

PDZD7 c.490C>T, p.R164W *PDZD7* c.1574_1597del, p.525_533del

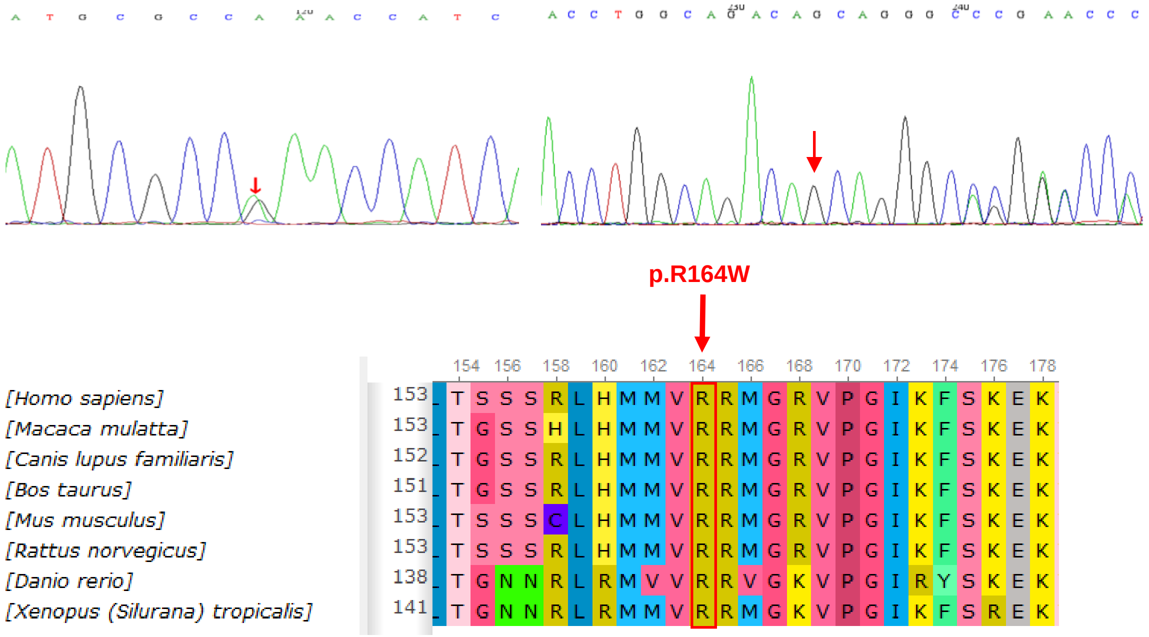

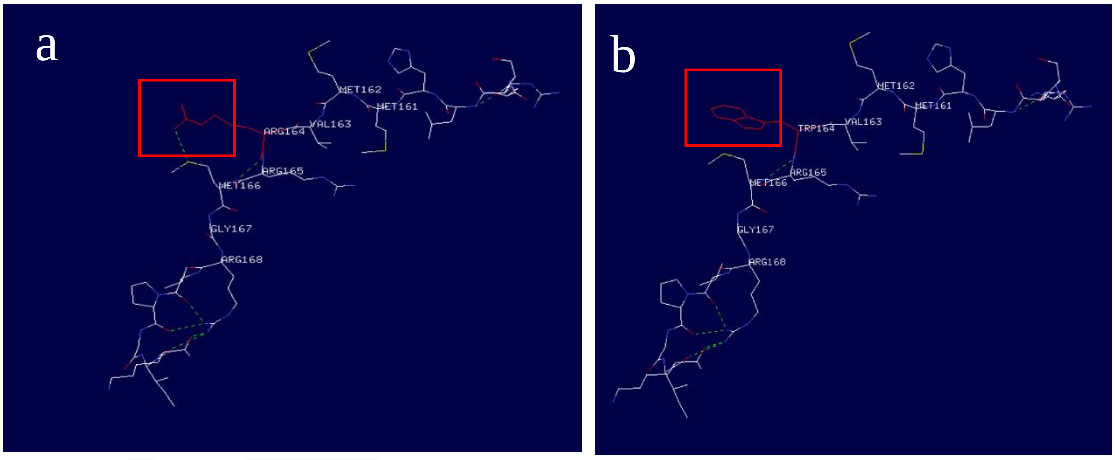

Wild（a）, Mutation p.R164W（b）: A hydrogen bond between 164 and 166 were broken after the 164 Trp mutation.

PDZD7(c.1574_1597delACCAGGAGAGGGGCCGGGCCCTGC, p.525_533delDQERGRALLinsV)

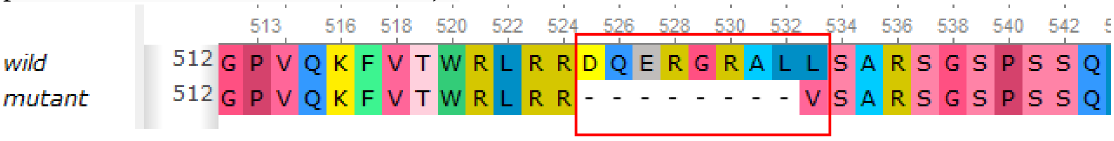

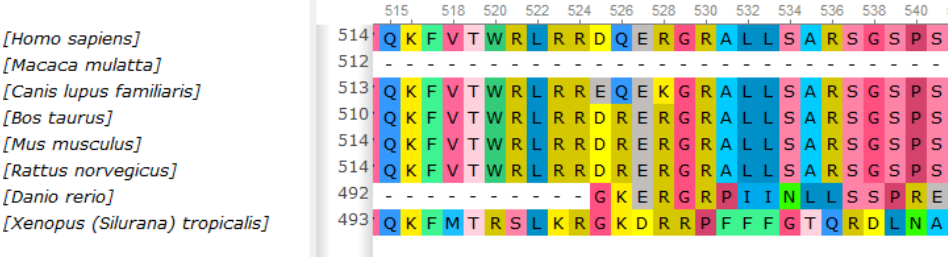

There is no protein structural homology-model for p.525_533delDQERGRALLinsV.

NT-48-II:5（F/47Y）

KARS c.685T>C, p.Y229H

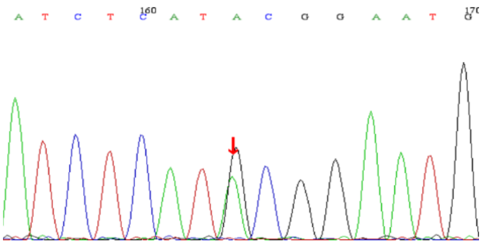

KARS c.403G>A, p.D135N

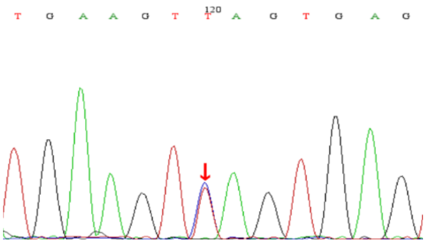

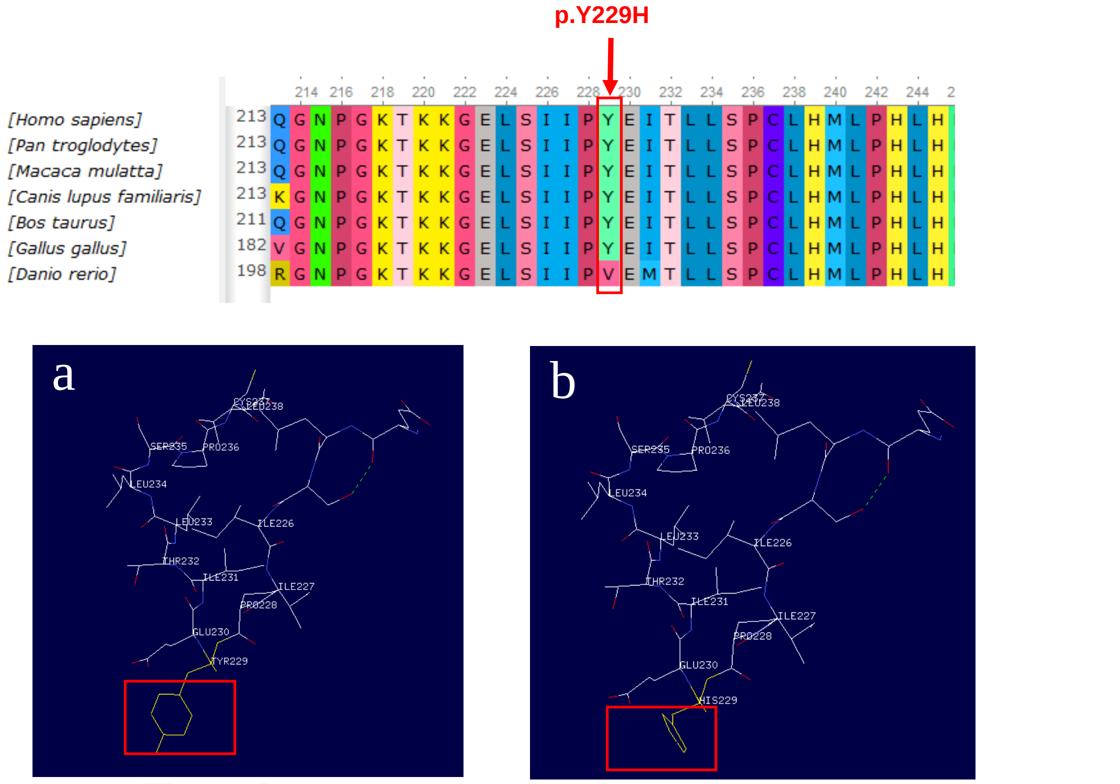

Wild（a），Mutation p.Y229H（b）: Nothing else changed expect the change p.Y229H.

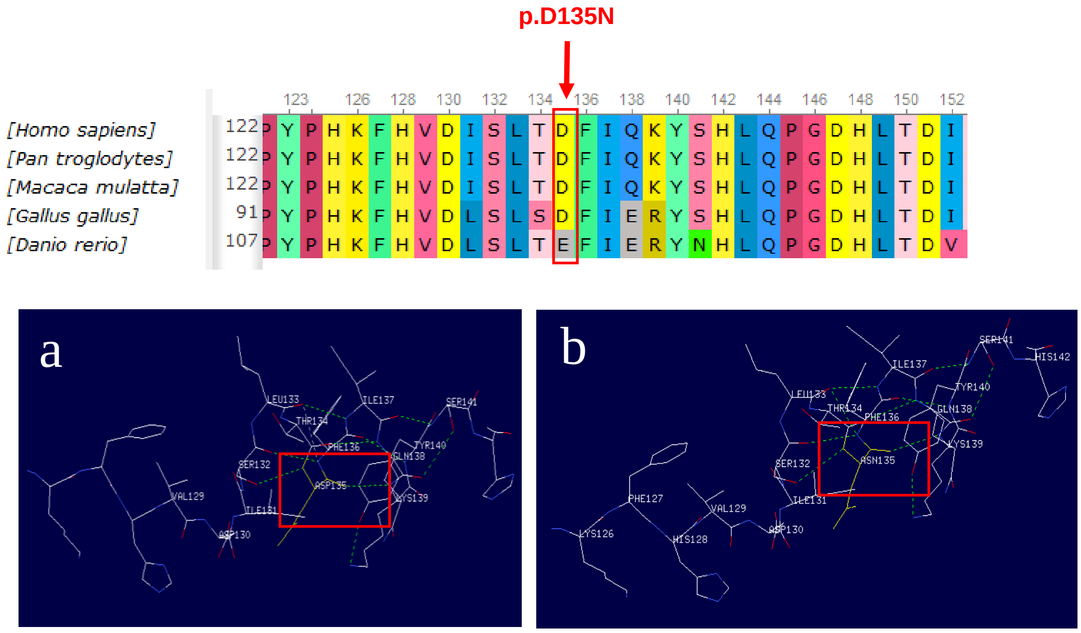

Wild（a），Mutation p.D135N（b）: Nothing else changed expect the change p.D135N.

NT-51-II:1（F/30Y）

OTOG c.433G>A, p.G145S

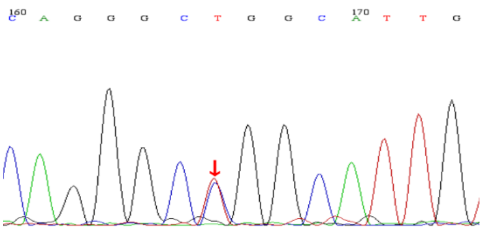

OTOG c.2117-6C>T

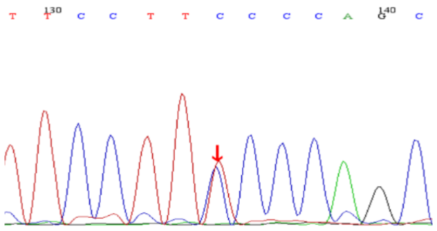

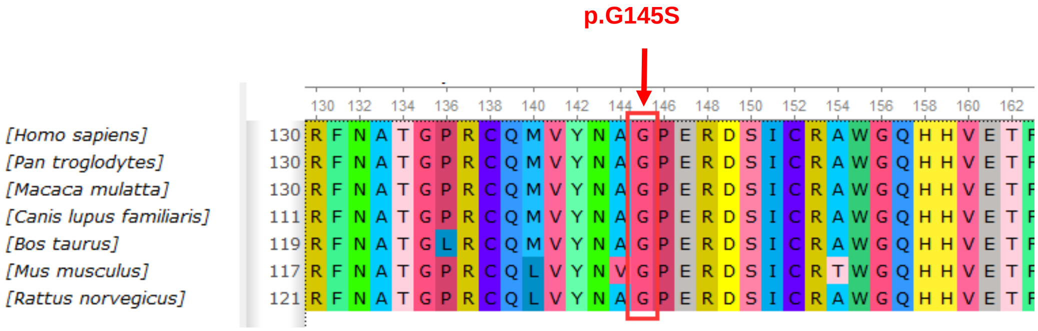

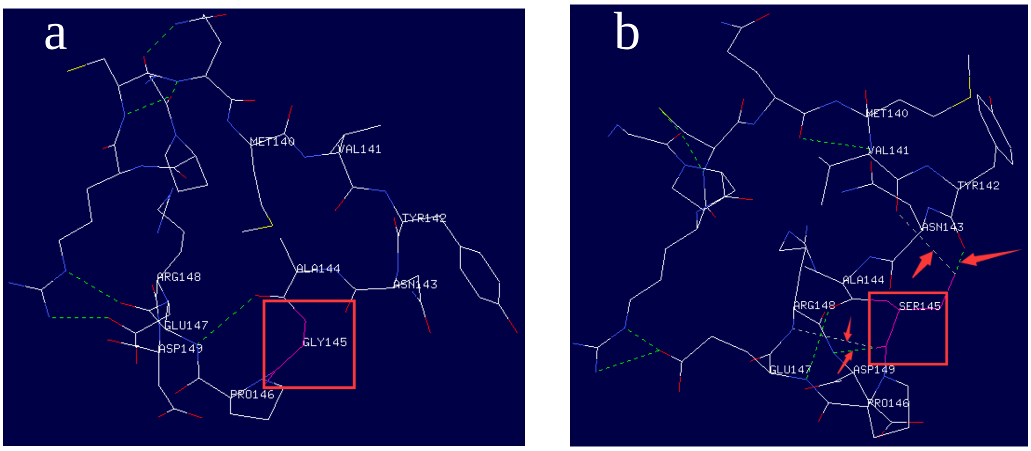

Wild（a）, Mutation p.G145S（b）: Four hydrogen bonds were formed among 145 and 141, 142,148,149, respectively after the mutation.

NT-52-II:3（F/72Y）

GRXCR2 c.65A >G, p.K22R

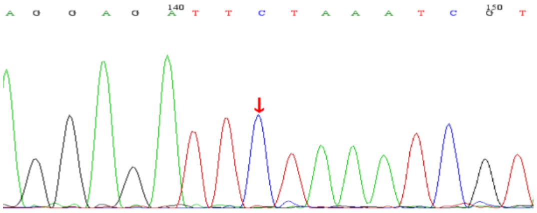

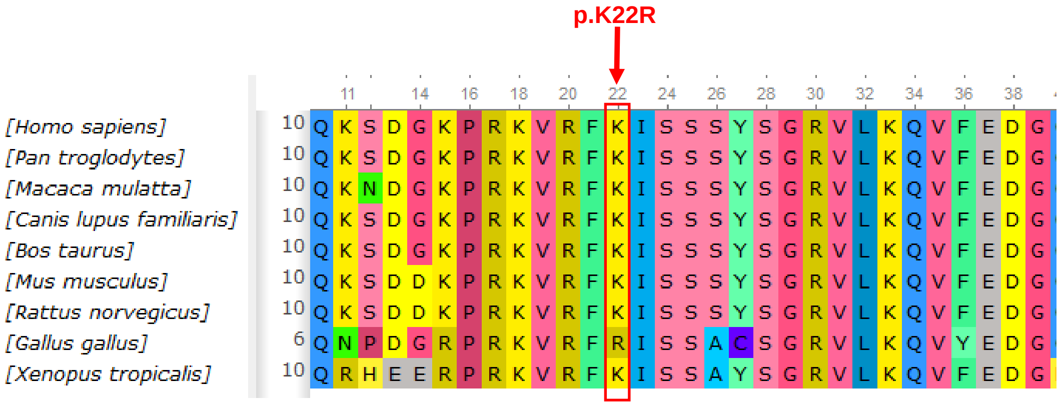

There is no protein structural homology-model for p.K22R.
